# Habitual Fructose Intake Relates to Insulin Sensitivity and Fatty Liver Index in Recent-Onset Type 2 Diabetes Patients and Individuals without Diabetes

**DOI:** 10.3390/nu10060774

**Published:** 2018-06-15

**Authors:** Katharina S. Weber, Marie-Christine Simon, Klaus Strassburger, Daniel F. Markgraf, Anette E. Buyken, Julia Szendroedi, Karsten Müssig, Michael Roden, GDS Group

**Affiliations:** 1 Institute for Clinical Diabetology, German Diabetes Center at Heinrich Heine University, Leibniz Institute for Diabetes Research, 40225 Düsseldorf, Germany; katharina.weber@ddz.uni-duesseldorf.de (K.S.W.); marie-christine.simon@uni-bonn.de (M.-C.S.); daniel.markgraf@ddz.uni-duesseldorf.de (D.F.M.); julia.szendroedi@ddz.uni-duesseldorf.de (J.S.); karsten.muessig@ddz.uni-duesseldorf.de (K.M.); 2 German Center for Diabetes Research (DZD), 85764 München-Neuherberg, Germany; klaus.strassburger@ddz.uni-duesseldorf.de; 3 Institute for Biometrics and Epidemiology, German Diabetes Center at Heinrich Heine University, Leibniz Institute for Diabetes Research, 40225 Düsseldorf, Germany; 4 Institute of Nutrition, Consumption and Health, Faculty of Natural Sciences, University Paderborn, 33098 Paderborn, Germany; anette.buyken@uni-paderborn.de; 5 Division of Endocrinology and Diabetology, Medical Faculty, Heinrich Heine University, 40225 Düsseldorf, Germany

**Keywords:** dietary fructose, peripheral insulin sensitivity, hepatic insulin sensitivity, observational cohort study

## Abstract

The association between the amount and sources of fructose intake with insulin sensitivity and liver fat needs further elucidation. This study aimed at examining whether habitual intake of sucrose plus non-sucrose bound as well as of non-sucrose bound fructose (total fructose, fruit-derived, juice-derived, sugar sweetened beverages (SSB)-derived fructose) is cross-sectionally associated with insulin sensitivity and fatty liver index (FLI). Fructose intake was estimated using the EPIC food frequency questionnaire from 161 participants with type 2 diabetes (T2D) in the ongoing German Diabetes Study (GDS) (age 53 ± 9 years; HbA1c 6.4 ± 0.9%) and 62 individuals without diabetes (CON) (47 ± 14 years; 5.3 ± 0.3%). Peripheral (M-value) and hepatic insulin resistance were assessed by hyperinsulinemic-euglycemic clamps with stable isotope dilution. FLI was calculated based on body mass index, waist circumference, triglyceride and gamma glutamyl transferase concentrations. Multivariable linear regression analyses were performed. A doubling of SSB-derived sucrose plus non-sucrose bound as well as of non-sucrose bound fructose intake was independently associated with a reduction of the M-value by −2.6% (−4.9; −0.2) and −2.7% (−5.2; −0.1) among T2D, respectively, with an increase in the odds of fatty liver by 16% and 17%, respectively among T2D (all *p* < 0.05). Doubling fruit-derived sucrose plus non-sucrose bound fructose intake independently related to a reduction in the odds of fatty liver by 13% (*p* = 0.033) among T2D. Moderate SSB-derived fructose intake may detrimentally affect peripheral insulin sensitivity, whereas fruit-derived fructose intake appeared beneficial for liver fat content.

## 1. Introduction

There is increasing concern that dietary fructose may be a key contributor to the rising prevalence of metabolic disorders [1]. Fructose intake may specifically affect the liver due to its mainly hepatic metabolism [2]. In individuals without diabetes, the addition of fructose to diets in isocaloric exchange for other macronutrients does not affect hepatic fat content and insulin sensitivity [3,4]. However, fructose intake exceeding 250 g/day and providing an excess of calories may reduce hepatic insulin sensitivity in healthy, obese or genetically at-risk individuals for diabetes [4]. Fructose overconsumption increased hepatocellular fat content in some, but not all intervention studies [5,6]. Thus, the effect of fructose intake on hepatic insulin sensitivity and hepatocellular fat content seems to critically depend on its energy contribution to the diet. However, the role of moderate or high fructose intake on peripheral insulin sensitivity remains uncertain [4].

In addition to the amount of fructose, its source might also influence its metabolic effects. According to a cross-sectional population-based survey among genetically susceptible individuals for type 2 diabetes mellitus (T2D), total dietary fructose and fructose from fruit juices were associated with an increased risk for glucose intolerance, while fructose from fresh fruits were related to a lower risk for T2D [7]. Also, consumption of sugar-sweetened beverages (SSB) has been linked to unfavorable metabolic outcomes [8], and specifically, overconsumption of fructose-sweetened beverages compared to glucose-sweetened beverages decreased insulin sensitivity among overweight and obese individuals without diabetes [9]. Thus, the relationship between moderate amounts of dietary fructose from different sources with tissue-specific insulin sensitivity and liver fat content among persons with T2D requires further elucidation.

This study tested the hypothesis that higher habitual intake of fructose from regular food sources (assessed as total fructose, fructose from fruits, fructose from juices, and fructose from SSB) are associated with lower hepatic but not peripheral insulin sensitivity and higher fatty liver index (FLI) in patients with recently diagnosed T2D. Additionally, the study aims to examine whether this can also be observed in individuals without diabetes (CON).

## 2. Materials and Methods

### 2.1. Study Population

Participants were recruited from the ongoing German Diabetes Study (GDS; clinicaltrials.gov: NCT01055093), a prospective observational cohort study investigating the natural history of diabetes and the development of diabetes-related comorbidities as described in detail elsewhere [10]. Individuals with T2D are patients with recently diagnosed type 2 diabetes mellitus (time since diagnosis <12 months), while CON are glucose tolerant individuals based on a standardized 75-g oral glucose tolerance test [11] and have no first- or second-degree relatives with diabetes. For the present cross-sectional analyses of baseline data, patients with T2D and CON were included consecutively between August 2012 and June 2016, if they provided data on their habitual food intake and underwent the hyperinsulinemic-eugylcemic clamp test.

### 2.2. Ethics

All participants gave their written informed consent to the study, which was approved by the ethics committee of the Heinrich Heine University Düsseldorf, Germany, and is performed according to the most recent version of the Declaration of Helsinki.

### 2.3. Nutrition Assessment

Habitual dietary intake was assessed using the semi-quantitative food frequency questionnaire (FFQ), which was designed and validated within the European Prospective Investigation into Cancer and Nutrition (EPIC)-Potsdam study [10,12]. Briefly, the EPIC-FFQ asks for usual food consumption frequencies of 148 food items within the last twelve months, for an average portion size. The main sources of fructose investigated in the questionnaire are fruits, SSB, vegetables, juices, sugar and confectionary, cake, and alcoholic beverages. Within the EPIC study, the relative validity of the EPIC-FFQ has been assessed by comparison with repeatedly collected 24 h dietary recalls for total carbohydrate, disaccharide, and monosaccharide intake with classification into the same or adjacent quintiles of 70% for monosaccharides [13]. Within the GDS, the questionnaire was applied at the time of inclusion in the study, i.e., within the first year after diabetes diagnosis. Mean total daily energy (TEI) (MJ/day) and fructose (g/day) intake was derived for each participant. For the present analysis, fructose intake (g/day) was considered as sucrose plus non-sucrose bound fructose and as non-sucrose bound fructose intake, and further differentiated into total fructose intake, fructose intake from fruits, fructose intake from juices, and fructose intake from SSB.

### 2.4. Anthropometric and Laboratory Analyses

Anthropometric measures (i.e., body mass index (BMI)) and parameters of clinical chemistry (i.e., fasting blood glucose, fasting insulin, HbA1c, GGT) were assessed as previously described [10]. FLI was calculated based on BMI, waist circumference, serum triglyceride and gamma glutamyl transferase (GGT) concentrations as described before [14].

### 2.5. Whole-Body (Peripheral) and Hepatic Insulin Sensitivity

The hyperinsulinemic-euglycemic clamp as part of the Botnia-clamp protocol was used to assess peripheral insulin sensitivity, which was expressed as whole-body glucose disposal (M-value), i.e., space-corrected mean glucose infusion rate during the last 30 min of the clamp [10]. In a subgroup (*n* = 113), data on endogenous glucose production were available. Endogenous glucose production was measured during the hyperinsulinemic-euglycemic clamp test using continuous infusion of d-[6,6^2^H_2_]glucose [10] to assess the hepatic insulin resistance index from the product of basal endogenous glucose production and fasting insulin concentration [15]. Hepatic insulin resistance index was not calculated for patients treated with intermediate- or long-acting insulin (*n* = 3), as the insulin dose applied in the evening preceding the examination might affect the fasting insulin concentrations.

### 2.6. Socio-Economic Status

Marital status, current employment status, and highest school-leaving qualification [16,17] were assessed from standardized questionnaires as single dimensions of the socio-economic status (SES).

### 2.7. Physical Activity

Physical activity was assessed by a standardized international questionnaire, which has also been validated in the German population [10,18,19]. For the present analysis, leisure time physical activity and sports were considered as single dimensions of physical activity. Intensity of leisure time physical activity and sports were classified using a standard manual in order to obtain weighting factors for calculation of a leisure time activity and sports index [10,19].

### 2.8. Statistics

To improve normality, TEI, fructose intake from fruits, juices, fruits and SSB, M-value, and hepatic insulin resistance index were ln-transformed prior to analysis. Additionally, the logit transformation (logit(*x*) = ln(*x*/1 − *x*) was applied to FLI, divided by 100, to obtain a corresponding linear index [20].

Fructose intake from fruits, fructose intake from juices, and fructose intake from SSB (ln-transformed) were adjusted for TEI (ln-transformed) using the residual method [21] separately for males and females with and without T2D.

Differences between patients with T2D and CON were tested using Student’s *t*-test for continuous variables with ln-transformation of non-normally distributed variables and Fisher’s exact test for categorical variables.

Multivariable linear regression models were applied with fructose intake as independent variable, which was adjusted for TEI [residual], and M-value, FLI, and hepatic insulin resistance index as dependent variables. Associations for fructose intake from the three different sources were calculated in separate multivariable linear regression models. For presentation in tables and figures, regression coefficients were re-transformed to relative changes [%] with a doubling of fructose intake. As the FLI expresses the estimated probability of developing a fatty liver [14], the re-transformed regression coefficients represent the change in the odds of having a fatty liver with a doubling of fructose intake.

Model 1 was adjusted for TEI (MJ), metabolic status [T2D, CON], age, sex and BMI and also included an interaction term between metabolic status and sex as well as an interaction between subject group (T2D and CON) and fructose intake. As the BMI possibly mediates the association between fructose intake from SSB and insulin sensitivity and FLI [22,23,24], models with fructose from SSB as an independent variable were not adjusted for this variable. Model 2 was additionally adjusted for marital status (with spouse/unmated), highest school-leaving qualification (advanced technical college certificate/high school graduation: yes/no), and current employment status (employed/unemployed) and model 3 also considered leisure time physical activity and the sports index as potential confounding factors.

The PROC POWER procedure for multivariable linear regression was applied for power and sample size analyses in SAS [25]. A sample size of *n* = 323 ensures that an association between M-value, FLI and fructose intake (total fructose intake, fructose intake from fruits, fructose intake from juices, fructose intake from SSB) can be detected with a power of 80% if the corresponding partial correlation adjusted for up to eight potential confounders is greater than or equal to 0.16. For the subgroup analysis (*n* = 110), associations between hepatic insulin resistance index and fructose intake can be detected with a power of 80% if the corresponding partial correlation adjusted for up to eight potential confounders is greater than or equal to 0.27.

Statistical analyses were carried out using SAS (version 9.4; SAS Institute, Cary, NC, USA). *p*-values < 0.05 were considered statistically significant.

## 3. Results

### 3.1. Volunteers’ Characteristics

In total, 223 participants (161 patients with recently diagnosed T2D and 62 CON) were included in the analyses (Figure S1). Patients with T2D were older and had higher BMI and FLI as well as worse glycemia and lower peripheral and hepatic insulin sensitivity than CON (Table 1). While total carbohydrate, disaccharide, sucrose and sucrose plus non-sucrose bound fructose intake was higher among CON compared to T2D, total non-sucrose bound fructose intake was comparable between groups (Table 2). Sucrose plus non-sucrose bound fructose intake from fruits accounted for about 29% of total fructose intake, while fructose intake from SSB accounted for about 10% and fructose intake from juices for about 8% of total fructose intake in this cohort.

### 3.2. Association between Intake of Fructose and Peripheral Insulin Sensitivity

**Figure 1:**
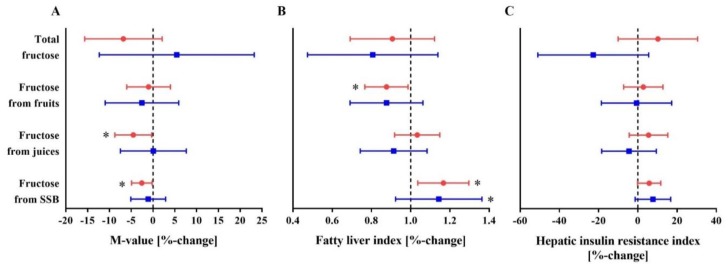
Association of sucrose plus non-sucrose bound total fructose, fructose from fruits, fruit juices and sugar-sweetened beverages with peripheral insulin sensitivity (M-value) (**A**), fatty liver index (**B**) and hepatic insulin resistance index (**C**) in patients with type 2 diabetes (T2D) and individuals without diabetes (CON). Data are summarized as relative changes, 95% confidence intervals (**A**,**C**) or odds ratios, 95% confidence intervals (**B**) and *p*-values from linear regression analyses, calculated using the M-value (**A**), the fatty liver index (**B**) and the hepatic insulin resistance index (**C**) as outcome variables. Total fructose as well as fructose from fruits, fruit juices and sugar-sweetened beverages were defined as exposure variables for linear regression analyses with fructose intake adjusted for the total daily energy intake [MJ] using the residual method. Red circles indicate individuals with type 2 diabetes, blue squares indicate individuals without diabetes (CON). * *p* < 0.05. Regression analyses are adjusted for total daily energy intake, age, BMI (not for models using fructose from SSB as independent variable), metabolic status, sex, the interaction term between metabolic status and sex, an interaction between subject group (T2D and CON) and fructose intake, leisure time physical activity and sports, marital status, highest school-leaving qualification, and current employment status. Relative changes (95% CI) (**A**,**C**) should be interpreted as follows: a doubling of fructose intake associates with a %-change of M-value (**A**), hepatic insulin resistance index (**C**) by the respective relative change. Odds ratios (95% CI) (**B**) should be interpreted as follows: a doubling of fructose intake associates with a change in the odds of having a fatty liver by the respective odds ratio. SSB, sugar-sweetened beverages.

Higher energy-adjusted intake of sucrose plus non-sucrose bound as well as of non-sucrose bound fructose from juices was associated with lower M-value among patients with T2D, but not among CON. Also, higher energy-adjusted intake of sucrose plus non-sucrose bound as well as of non-sucrose bound fructose from SSB related to lower M-value among patients with T2D, but not among CON (Figure 1A, Table 3). Intake of sucrose plus non-sucrose bound as well as of non-sucrose bound total fructose and of fructose from fruits was not independently related to M-value (Table 3).

### 3.3. Association between Intake of Fructose and FLI

Among patients with T2D, each doubling of sucrose plus non-sucrose bound fructose intake from fruits at a constant TEI level was related to a reduction in the odds of having a fatty liver by 13% (*p* = 0.023) (model 3) (Figure 1B, Table 4). For non-sucrose bound fructose intake, this association was slightly attenuated after adjustment for the parameters of physical activity (odds of having a fatty liver was reduced by 11% (*p* = 0.056, model 3)) (Table 4). Each doubling of sucrose plus non-sucrose bound and of non-sucrose bound fructose intake from SSB was associated with an increase in the odds of having a fatty liver based on the FLI by 16% and 17% among individuals with T2D, respectively (Figure 1B, Table 4, model 3). Intake of sucrose plus non-sucrose bound as well as of non-sucrose bound total fructose and of fructose from fruit juices was not independently associated with FLI (Table 4).

### 3.4. Association between Intake of Fructose and Hepatic Insulin Resistance Index

Within a subgroup of individuals with data on hepatic insulin resistance index (*n* = 105, Figure S1), higher energy-adjusted intake of sucrose plus non-sucrose bound as well as of non-sucrose bound fructose from SSB was associated with higher hepatic insulin resistance index among T2D and CON (Table 5, model 2). However, this association was attenuated after adjustment for parameters of physical activity (Figure 1C, Table 5, model 3). Neither intake of sucrose plus non-sucrose bound nor of non-sucrose bound total fructose or fructose from fruits or fruit juices was independently associated with hepatic insulin resistance (Table 5).

## 4. Discussion

This study indicates that habitually consumed amounts of fructose from fruits are inversely associated with the odds of having a fatty liver among T2D, while higher intake of fructose from SSB is related to higher peripheral insulin resistance and higher odds of having a fatty liver among T2D. 

### 4.1. Association between SSB-Derived Non-Sucrose Bound Fructose Intake and Peripheral Insulin Sensitivity

The observed association between higher SSB-derived fructose intake and lower M-value among T2D in our cohort is comparable to the findings of two previous intervention studies, reporting that a higher fructose intake, administered as fructose-sweetened beverages or fructose powder dissolved in water, reduced peripheral insulin sensitivity as derived from the oral glucose tolerance test and the deuterated-glucose disposal test. In contrast to the present study, the fructose intake in the two intervention studies, which were conducted on individuals without diabetes, markedly exceeded habitual dietary intakes, i.e., 25% of daily energy requirement and 150 g/day [9,26]. Of note, we already observed the inverse association between fructose from SSB and the M-value at considerably lower fructose intake levels from SSB of about 0.4 g/day, which may be due to the recruitment of insulin resistant individuals with T2D.

Interestingly, the present study found evidence for the detrimental effect of moderate fructose consumption on peripheral insulin sensitivity. Although fructose intake has been assumed to specifically affect the liver due to its mainly hepatic metabolism [2], elevated fructose intake habitually consumed over longer periods of time might additionally modify peripheral insulin sensitivity [27]. The underlying mechanism could involve progressive fat deposition in skeletal muscle as a result secondary to excessive hepatic de novo lipogenesis and secretion of very low density lipoproteins, ultimately exceeding the capacity of the liver to store triglycerides [28]. As a result, the increased flux of fatty acids towards muscle leads to the accumulation of fat and lipotoxic lipid metabolites, which in turn induce insulin resistance [29]. In line with this contention, the present study confirmed a higher frequency of hepatic steatosis among the insulin-resistant T2D compared to insulin-sensitive CON.

The proposed detrimental effects of fructose compared to glucose are attributed to (i) its mainly hepatic metabolism [2]; (ii) it serves as a substrate for de novo lipogenesis, thereby contributing to hepatic triglyceride synthesis and accumulation [30,31]; (iii) its insulin-independent metabolization promoting excessive lipid formation and deposition [32]; (iv) its non-enzymatic “fructation” and reactive oxygen species formation related to cellular dysfunction [33]; and (v) its failure to suppress the hunger hormone ghrelin, potentially resulting in dietary overconsumption [34].

### 4.2. Association between Fruit-Derived Non-Sucrose Bound Fructose Intake and Fatty Liver Index

In our cohort, fruits were by far the most relevant source of fructose, providing about 8 g of fructose per day. While short-term fructose overfeeding might increase hepatic fat content [35], the role of habitual intake of fructose from fruits on liver fat content is unknown. In the present study, a doubling of the intake of fruit-derived fructose was associated with a decrease in the odds of having a fatty liver among T2D, suggesting that the source of fructose is important in determining the risk for metabolic alterations. Of note, this association did not seem to be mediated by energy intake. An inverse association has been previously observed between fructose intake and glycemic control, suggesting that low-dose fructose intake, i.e., ≤36 g/day, may improve HbA1c among T2D [36]. A comparable improvement in HbA1c has also been achieved by an increase in fruit consumption with a low glycemic index, which provided a similar low-dose increase in fructose intake [37]. Thus, our results support the beneficial role of fruits in the diet of individuals with T2D, which might be additionally mediated by (soluble) dietary fiber, vitamins, flavonoids, and antioxidants [38] provided by fruits. In this case, the present inverse association between fruit-derived fructose intake and the odds of having a fatty liver might be explained by residual confounding. Fructose ingestion from fruits would thus be a proxy for the above listed ingredients of fruits.

### 4.3. Sucrose Plus Non-Sucrose Bound Fructose Intake vs. Non-Sucrose Bound Fructose Intake

In parallel with the mechanistic studies assessing the role of pure fructose on metabolism [8], we analyzed the association of non-sucrose bound fructose with insulin sensitivity and fatty liver index. However, being aware of the criticism expressed about these mechanistic studies and the fact that fructose is always consumed together with glucose [39], we also conducted analyses for sucrose plus non-sucrose bound total fructose intake.

### 4.4. Strengths and Limitations

This study used gold standard methods, i.e., the hyperinsulinemic-euglycemic clamp with stable isotope dilution, to assess peripheral and hepatic insulin sensitivity [40]. Furthermore, including both insulin-sensitive and insulin-resistant humans allowed us to identify diseases-specific differences in the associations with fructose. Limitations comprise the observational study design and the smaller number of CON compared to patients with T2D. Residual confounding by e.g., dietary or lifestyle factors associated with the consumption of fruits, juices, and specifically SSB, needs to be considered when interpreting the present findings. Finally, hepatocellular fat content was assessed by the FLI rather than by magnetic resonance spectroscopy [41]. However, the FLI offers an acceptable diagnostic accuracy for larger studies in both individuals without diabetes [20] and patients with T2D [42].

## 5. Conclusions

The present study indicates that intake of fructose from SSB appears to have a detrimental effect on peripheral and hepatic insulin sensitivity, even when consumed in very moderate daily amounts. By contrast, fruit-derived fructose was not related to unfavorable metabolic effects and even showed inverse associations with the hepatocellular fat content of individuals with T2D.

## Figures and Tables

**Table 1:** Characteristics of patients with type 2 diabetes and individuals without diabetes.

	Type 2 Diabetes	CON	*p*
*N* (% males)	161 (68%)	62 (71%)	0.748
Age (years)	53.2 ± 9.1	46.5 ± 14.0	**<0.001**
Diabetes duration (months)	5.9 ± 3.2	-	-
Glucose-lowering medication [diet/oral glucose-lowering medication/oral glucose-lowering medication + insulin/insulin]	55 (34%)/(61%)/4 (2%)/4 (2%)	-	-
BMI (kg/m^2^)	32.1 ± 6.0	28.0 ± 5.6	**<0.001**
Waist circumference (cm)	107 ± 15	95 ± 17	**<0.001**
Fasting blood glucose (mg/dL)	130 ± 29	92 ± 16	**<0.001**
Fasting insulin (mU/L)	17.7 (13.2; 24.8)	7.8 (5.3; 11.6)	**<0.001**
HbA1c (% (mmol/mol))	6.4 ± 0.9 (46.5 ± 9.7)	5.3 ± 0.3 (33.9 ± 3.1)	**<0.001**
M-value (body weight, with space correction) (mg∙kg^−1^∙min^−1^)	5.4 (4.2; 7.5)	10.3 (8.5; 12.3)	**<0.001**
Hepatic insulin resistance index (mg∙kg^−1^∙min^−1^∙mU^−1^∙L) *	31.8 (21.5; 40.9)	17.9 (11.4; 23.6)	**<0.001**
Fatty liver index [a.u.]	84.6 (62.5; 94.7)	36.5 (12.5; 73.1)	**<0.001**
Marital status [with spouse/unmated]	123 (76%)/38 (24%)	44 (71%)/18 (29%)	0.395
Highest school-leaving qualification: advanced technical college certificate/high school graduation [yes/no]	87 (54%)/74 (46%)	40 (65%)/22 (35%)	0.176
Current employment status [employed/unemployed]	127 (79%)/34 (21%)	41 (66%)/21 (34%)	0.057

Data are *n* (%), mean ± SD or median (P_25_; P_75_). Data only available for * *n* = 73 patients with type 2 diabetes and *n* = 32 healthy controls. ^†^Fructose intake was adjusted for total energy intake using the residual method. Bold indicates *p* < 0.05. CON, individuals without diabetes.

**Table 2:** Dietary characteristics of patients with type 2 diabetes and individuals without diabetes.

	T2D	CON	*p*
**TEI** (MJ/day)	8.9 (7.1; 11.7)	10.0 (8.0; 12.6)	**0.029**
**Total sucrose plus non-sucrose bound fructose**			
(g/day)	40.2 (30.5; 59.4)	47.9 (38.2; 69.0)	**0.016**
(% of TEI)	7.9 (6.0; 10.5)	8.9 (6.9; 10.9)	0.232
**Sucrose plus non-sucrose bound fructose from fruits**			
(g/day)	11.0 (6.4; 18.9)	12.3 (7.5; 17.6)	0.924
(% of TEI)	2.1 (1.2; 3.3)	2.2 (1.2; 2.9)	0.404
**Sucrose plus non-sucrose bound fructose from juices**			
(g/day)	1.6 (1.1; 3.6)	2.5 (1.3; 5.4)	**0.029**
(% of TEI)	0.3 (0.2; 0.6)	0.4 (0.3; 0.9)	0.146
**Sucrose plus non-sucrose bound fructose from SSB**			
(g/day)	0.53 (0.21; 1.81)	0.53 (0.21; 5.79)	0.102
(% of TEI)	0.09 (0.05; 0.48)	0.15 (0.05; 0.88)	0.186
**Total non-sucrose bound fructose**			
(g/day)	19.6 (13.3; 28.6)	21.4 (14.6; 32.5)	0.279
(% of TEI)	3.6 (2.7; 5.4)	3.4 (2.9; 4.7)	0.850
**Non-sucrose bound fructose from fruits**			
(g/day)	7.2 (4.0; 13.8)	8.5 (4.6; 11.2)	0.759
(% of TEI)	1.4 (0.8; 2.4)	1.3 (0.8; 1.9)	0.253
**Non-sucrose bound fructose from juices**			
(g/day)	0.8 (0.5; 1.9)	1.3 (0.6; 3.0)	**0.034**
(% of TEI)	0.2 (0.1; 0.3)	0.2 (0.1; 0.4)	0.133
**Non-sucrose bound fructose from sugar-sweetened beverages**			
(g/day)	0.4 (0.2; 1.2)	0.4 (0.2; 3.8)	0.110
(% of TEI)	0.07 (0.04; 0.33)	0.10 (0.05; 0.58)	0.209
**Carbohydrates**			
(g/day)	199.4 (148.1; 255.8)	214.7 (173.3; 306.5)	**0.021**
(% of TEI)	37.9 (33.3; 41.4)	39.4 (35.0; 43.1)	0.244
**Disaccharides**			
(g/day)	56.3 (40.3; 74.9)	68.3 (53.4; 97.1)	**<0.001**
(% of TEI)	10.6 (8.5; 13.5)	12.5 (9.3; 15.1)	**0.009**
**Sucrose**			
(g/day)	40.8 (30.5; 57.4)	54.8 (40.9; 76.9)	**<0.001**
(% of TEI)	8.2 (6.4; 10.8)	10.1 (70; 12.3)	**0.009**

Data are median (P_25_; P_75_). Bold indicates *p* < 0.05. CON, individuals without diabetes; TEI, total daily energy intake; T2D, individuals with type 2 diabetes.

**Table 3:** Association of fructose intake with peripheral insulin sensitivity (M-value) in patients with type 2 diabetes and individuals without diabetes.

Parameter/Model	T2D (*n* = 161)		CON (*n* = 62)	
	Relative Change (95% CI) *	*p*	Relative Change (95% CI) *	*p*
*Sucrose plus non-sucrose bound fructose*				
Total fructose [residual]				
Model 1	−5.9 (−14.6; 3.7)	0.219	−0.9 (−16.5; 17.5)	0.915
Model 2	−6.7 (−15.3; 2.9)	0.164	1.7 (−14.2; 20.7)	0.844
Model 3	−7.1 (−15.5; 2.3)	0.132	4.5 (−11.8; 23.7)	0.611
Fructose from fruits [residual]				
Model 1	0.3 (−4.6; 5.5)	0.912	0.6 (−7.6; 9.6)	0.886
Model 2	0.0 (−4.9; 5.1)	0.998	0.2 (−8.1; 9.2)	0.967
Model 3	−1.1 (−6.0; 4.1)	0.671	−2.8 (−10.8; 6)	0.523
Fructose from juices [residual]				
Model 1	−3.6 (−7.8; 0.8)	0.106	0.7 (−6.7; 8.8)	0.856
Model 2	−3.9 (−8.1; 0.5)	0.083	−0.2 (−7.6; 7.8)	0.963
Model 3	−4.6 (−8.7; −0.3)	**0.038**	−0.1 (−7.4; 7.7)	0.979
Fructose from SSB [residual]				
Model 1	−3.1 (−5.5; −0.7)	**0.011**	−2.5 (−6.4; 1.5)	0.218
Model 2	−2.9 (−5.3; −0.5)	**0.017**	−2.3 (−6.1; 1.8)	0.264
Model 3	−2.6 (−4.9; −0.2)	**0.035**	−1.1 (−5.1; 2.9)	0.575
*Non-sucrose bound fructose*				
Total fructose [residual]				
Model 1	−5.1 (−12.0; 2.3)	0.174	−1.6 (−14.3; 13.0)	0.816
Model 2	−5.9 (−12.8; 1.5)	0.112	0.1 (−12.9; 14.9)	0.990
Model 3	−6.4 (−13.1; 0.8)	0.081	2.1 (−11.0; 17.0)	0.767
Fructose from fruits [residual]				
Model 1	0.2 (−4.3; 4.9)	0.923	0.1 (−7.4; 8.3)	0.976
Model 2	−0.2 (−4.6; 4.5)	0.946	−0.4 (−7.9; 7.7)	0.912
Model 3	−1.1 (−5.6; 3.6)	0.627	−2.9 (−10.3; 5.0)	0.460
Fructose from juices [residual]				
Model 1	−3.5 (−7.1; 0.4)	0.076	0.3 (−6.3; 7.3)	0.931
Model 2	−3.7 (−7.4; 0.1)	0.058	−0.5 (−7.1; 6.5)	0.877
Model 3	−4.3 (−7.9; −0.5)	**0.025**	−0.5 (−6.9; 6.3)	0.875
Fructose from SSB [residual]				
Model 1	−3.3 (−5.9; −0.7)	**0.013**	−2.6 (−6.8; 1.7)	0.229
Model 2	−3.1 (−5.7; −0.5)	**0.019**	−2.3 (−6.5; 2.0)	0.282
Model 3	−2.7 (−5.2; −0.1)	**0.040**	−1.1 (−5.4; 3.3)	0.605

Data are relative changes, 95% confidence intervals (95% CI), and *p*-values from linear regression analyses, calculated using the M-value as outcome variable. Fructose intake was defined as exposure variable for linear regression analyses with fructose intake adjusted for the total daily energy intake [MJ] using the residual method. Fructose intake and M-value were entered into the models as ln-transformed variables. * Relative changes (95% CI) should be interpreted as follows: A doubling of fructose intake associates with a %-change of M-value by the respective relative change. Model 1 adjusted for total daily energy intake [MJ], age, BMI (not for models using fructose from SSB as independent variable), metabolic status, sex, the interaction term between metabolic status and sex as well as an interaction between subject group (T2D and CON) and fructose intake; Model 2 additionally adjusted for marital status, highest school-leaving qualification, and current employment status; Model 3 additionally adjusted for leisure time physical activity and sports index as potential confounding factor. Bold indicates *p* < 0.05. CON, individuals without diabetes; SSB, sugar-sweetened beverages; T2D, individuals with type 2 diabetes.

**Table 4:** Association of fructose intake with fatty liver index in patients with type 2 diabetes and individuals without diabetes.

Parameter/Model	T2D (*n* = 161)		CON (*n* = 62)	
	Relative Change (95% CI) *	*p*	Relative Change (95% CI) *	*p*
*Sucrose plus non-sucrose bound fructose*				
Total fructose [residual]				
Model 1	0.87 (0.68; 1.10)	0.245	0.85 (0.56; 1.30)	0.453
Model 2	0.89 (0.70; 1.14)	0.358	0.80 (0.53; 1.22)	0.309
Model 3	0.89 (0.70; 1.13)	0.354	0.76 (0.50; 1.16)	0.197
Fructose from fruits [residual]				
Model 1	0.85 (0.76; 0.96)	**0.010**	0.83 (0.68; 1.02)	0.082
Model 2	0.86 (0.76; 0.97)	**0.013**	0.85 (0.69; 1.04)	0.118
Model 3	0.87 (0.77; 0.99)	**0.033**	0.86 (0.70; 1.07)	0.167
Fructose from juices [residual]				
Model 1	1.01 (0.90; 1.12)	0.898	0.88 (0.73; 1.06)	0.178
Model 2	1.02 (0.91; 1.14)	0.720	0.90 (0.74; 1.09)	0.262
Model 3	1.03 (0.92; 1.15)	0.642	0.90 (0.75; 1.09)	0.284
Fructose from SSB [residual]				
Model 1	1.19 (1.06; 1.34)	**0.003**	1.18 (0.98; 1.42)	0.084
Model 2	1.18 (1.05; 1.33)	**0.005**	1.18 (0.97; 1.42)	0.093
Model 3	1.16 (1.04; 1.30)	**0.011**	1.13 (0.93; 1.37)	0.221
*Non-sucrose bound fructose*				
Total fructose [residual]				
Model 1	0.85 (0.71; 1.03)	0.095	0.82 (0.58; 1.15)	0.248
Model 2	0.88 (0.73; 1.05)	0.161	0.78 (0.56; 1.10)	0.156
Model 3	0.89 (0.74; 1.06)	0.195	0.75 (0.54; 1.06)	0.099
Fructose from fruits [residual]				
Model 1	0.87 (0.78; 0.97)	**0.016**	0.84 (0.69; 1.01)	0.063
Model 2	0.88 (0.79; 0.98)	**0.023**	0.85 (0.70; 1.03)	0.094
Model 3	0.89 (0.80; 1.00)	0.056	0.86 (0.71; 1.05)	0.142
Fructose from juices [residual]				
Model 1	1.01 (0.92; 1.11)	0.865	0.90 (0.76; 1.07)	0.227
Model 2	1.02 (0.93; 1.12)	0.688	0.92 (0.78; 1.09)	0.324
Model 3	1.03 (0.93; 1.13)	0.608	0.92 (0.78; 1.09)	0.347
Fructose from SSB [residual]				
Model 1	1.20 (1.06; 1.36)	**0.004**	1.20 (0.98; 1.47)	0.081
Model 2	1.19 (1.05; 1.35)	**0.006**	1.19 (0.97; 1.46)	0.091
Model 3	1.17 (1.03; 1.33)	**0.014**	1.14 (0.93; 1.40)	0.219

Data are odds ratios (OR), 95% confidence intervals (95% CI), and *p*-values from linear regression analyses, calculated based on the corresponding linear index of the fatty liver index (FLI) as outcome variable. Fructose intake was defined as the exposure variable for linear regression analyses with fructose intake adjusted for the total daily energy intake [MJ] using the residual method. Fructose intake was entered into the models as ln-transformed variables. * As the FLI expresses the estimated probability of developing a fatty liver (15), OR (95% CI) should be interpreted as follows: A doubling of fructose intake associates with a change in the odds of having a fatty liver by the respective OR. Model 1 adjusted for total daily energy intake [MJ], age, BMI (not for models using fructose from SSB as independent variable), metabolic status, sex, the interaction term between metabolic status and sex as well as an interaction between subject group (T2D and CON) and fructose intake; Model 2 additionally adjusted for marital status, highest school-leaving qualification, and current employment status. Model 3 additionally adjusted for leisure time physical activity and sports index as potential confounding factor. Bold indicates *p* < 0.05. CON, individuals without diabetes; OR, odds ratio; SSB, sugar-sweetened beverages; T2D, individuals with type 2 diabetes.

**Table 5:** Association of fructose intake with hepatic insulin resistance index in patients with type 2 diabetes and individuals without diabetes.

Parameter/Model	T2D (*n* = 73)		CON (*n* = 32)	
	Relative Change (95% CI) *	*p*	Relative Change (95% CI) *	*p*
*Sucrose plus non-sucrose bound fructose*				
Total fructose [residual]				
Model 1	6.0 (−12.9; 29.0)	0.559	−13.5 (−41.7; 28.4)	0.468
Model 2	6.8 (−12.5; 30.4)	0.513	−17.8 (−44.9; 22.8)	0.336
Model 3	9.0 (−9.4; 31.1)	0.356	−26.2 (−49.1; 7.1)	0.108
Fructose from fruits [residual]				
Model 1	−3.8 (−12.8; 6.2)	0.438	−10.8 (−25.5; 6.9)	0.212
Model 2	−3.5 (−12.6; 6.5)	0.474	−9.2 (−24.4; 9.0)	0.298
Model 3	2.5 (−7.1; 13.0)	0.621	−1.7 (−18; 17.8)	0.848
Fructose from juices [residual]				
Model 1	2.9 (−6.6; 13.3)	0.562	−5.6 (−18.9; 9.8)	0.451
Model 2	2.6 (−6.9; 13.1)	0.604	−5.8 (−19.6; 10.3)	0.453
Model 3	5.2 (−4.2; 15.5)	0.289	−5.2 (−18.0; 9.7)	0.471
Fructose from SSB [residual]				
Model 1	7.9 (2.2; 13.9)	**0.007**	10.8 (2.2; 20.2)	**0.013**
Model 2	7.6 (1.8; 13.7)	**0.010**	10.6 (1.8; 20.2)	**0.018**
Model 3	5.7 (−0.1; 11.7)	0.053	7.5 (−1.2; 16.9)	0.093
*Non-sucrose bound fructose*				
Total fructose [residual]				
Model 1	0.8 (−13.5; 17.4)	0.919	−2.2 (−28.6; 33.8)	0.8862
Model 2	1.6 (−12.9; 18.5)	0.839	−7.4 (−32.9; 27.9)	0.6384
	4.4 (−9.6; 20.5)	0.556	−15.0 (−37.2; 14.8)	0.285
Fructose from fruits [residual]				
Model 1	−3.3 (−11.6; 5.7)	0.450	−9.6 (−23.2; 6.3)	0.220
Model 2	−3.1 (−11.4; 6)	0.492	−8.4 (−22.3; 8)	0.292
	2.4 (−6.3; 11.8)	0.598	−1.5 (−16.3; 15.8)	0.851
Fructose from juices [residual]				
Model 1	3 (−5.5; 12.1)	0.501	−4.7 (−17; 9.4)	0.488
Model 2	2.7 (−5.8; 12)	0.537	−4.9 (−17.6; 9.7)	0.485
	5.1 (−3.2; 14.1)	0.235	−4.6 (−16.5; 8.9)	0.481
Fructose from SSB [residual]				
Model 1	8.3 (2.1; 14.8)	**0.008**	11.3 (2; 21.4)	**0.016**
Model 2	8.0 (1.7; 14.6)	**0.012**	11 (1.5; 21.4)	**0.023**
	5.9 (−0.3; 12.5)	0.061	7.6 (−1.7; 17.8)	0.112

Data are relative changes, 95% confidence intervals (95% CI), and *p*-values from linear regression analyses, calculated using the hepatic insulin resistance index as the outcome variable. Fructose intake was defined as the exposure variable for linear regression analyses with fructose intake as g/day as well as with fructose intake adjusted for the total daily energy intake [MJ] using the residual method. Fructose intake and hepatic insulin resistance index were entered into the models as ln-transformed variables. * Relative changes (95% CI) should be interpreted as follows: A doubling of fructose intake associates with a %-change of hepatic insulin resistance by the respective relative change. Model 1 adjusted for total daily energy intake [MJ], age, BMI (not for models using fructose from SSB as independent variable), metabolic status, sex, the interaction term between metabolic status and sex as well as an interaction between subject group (T2D and CON) and fructose intake; Model 2 additionally adjusted for marital status, highest school-leaving qualification, and current employment status; Model 3 additionally adjusted for leisure time physical activity and sports as potential confounding factor. Bold indicates *p* < 0.05. CON, individuals without diabetes; SSB, sugar-sweetened beverages; T2D, individuals with type 2 diabetes.

## Supplementary Materials

The following are available online at http://www.mdpi.com/2072-6643/10/6/774/s1. Figure S1: Flow diagram showing the number of participants included in the analyses from those enrolled in the German Diabetes Study.

## Abbreviations

BMI	body mass index
CON	individuals without diabetes
EPIC	European Prospective Investigation into Cancer and Nutrition
FFQ	food frequency questionnaire
FLI	fatty liver index
GGT	gamma glutamyl transferase
GDS	German Diabetes Study
M-value	whole-body glucose disposal
SES	socio-economic status
SSB	sugar-sweetened beverages
TEI	total daily energy intake
T2D	type 2 diabetes
