# Supplementary material for: Habitual Fructose Intake Relates to Insulin Sensitivity and Fatty Liver Index in Recent-Onset Type 2 Diabetes Patients and Individuals without Diabetes
Source: Nutrients. 2018 Jun 15;10(6):774. doi: 10.3390/nu10060774 (PMC6024554; doi:10.3390/nu10060774)
Supplement: Supplementary file 1 [file nutrients-10-00774-s001.pdf]

Supplementary material: Habitual Fructose Intake Relates to Insulin Sensitivity and Fatty Liver Index in Recent-Onset Type 2 Diabetes Patients and Individuals without Diabetes

Figure S1. Flow diagram showing the number of participants included in the analyses from those enrolled in the German Diabetes Study.

CON, individuals without diabetes. T2D, individuals with type 2 diabetes.

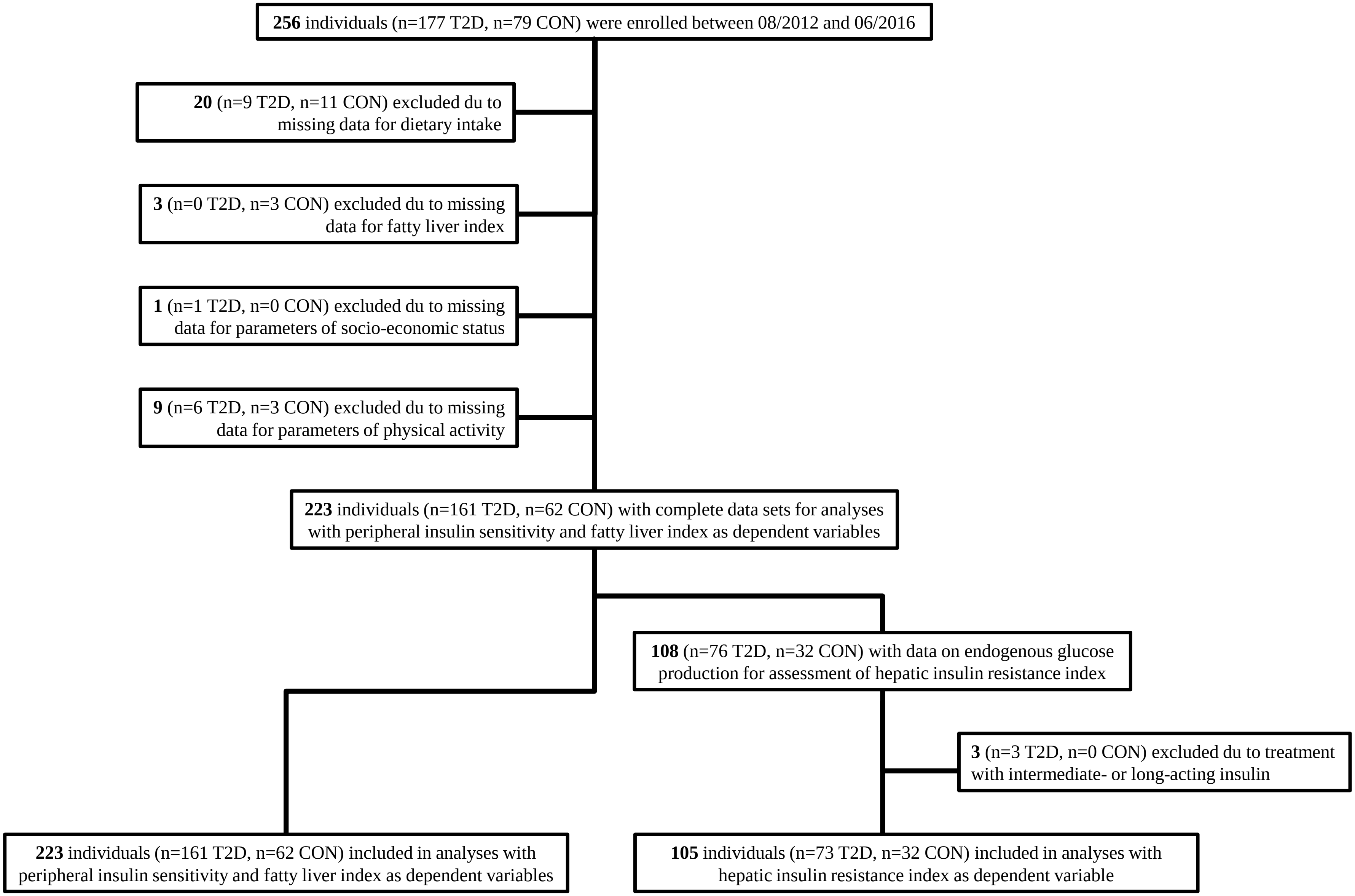

256 individuals (n=177 T2D, n=79 CON) were enrolled between 08/2012 and 06/2016
20 (n=9 T2D, n=11 CON) excluded du to missing data for dietary intake
3 (n=0 T2D, n=3 CON) excluded du to missing data for fatty liver index
1 (n=1 T2D, n=0 CON) excluded du to missing data for parameters of socio-economic status
9 (n=6 T2D, n=3 CON) excluded du to missing data for parameters of physical activity
223 individuals (n=161 T2D, n=62 CON) with complete data sets for analyses with peripheral insulin sensitivity and fatty liver index as dependent variables
108 (n=76 T2D, n=32 CON) with data on endogenous glucose production for assessment of hepatic insulin resistance index
3 (n=3 T2D, n=0 CON) excluded du to treatment with intermediate- or long-acting insulin
223 individuals (n=161 T2D, n=62 CON) included in analyses with peripheral insulin sensitivity and fatty liver index as dependent variables
105 individuals (n=73 T2D, n=32 CON) included in analyses with hepatic insulin resistance index as dependent variable
